# Onset of Ketosis-Prone Diabetes in the Setting of COVID-19 Infection

**DOI:** 10.7759/cureus.10779

**Published:** 2020-10-03

**Authors:** Raheel S Siddiqui, Milana Zirkiyeva, Merjona Saliaj

**Affiliations:** 1 Internal Medicine, Icahn School of Medicine at Mount Sinai (New York City Health and Hospitals/Queens), Jamaica, USA

**Keywords:** diabetic ketoacidosis (dka), ketosis prone diabetes, covid-19, diabetes type 2

## Abstract

Diabetic ketoacidosis is typically associated with type I diabetes mellitus, but it can be associated with type II diabetes mellitus under the conditions of extreme stress or as a presenting manifestation of ketosis-prone type II diabetes mellitus. A 38-year-old prediabetic male presented to the emergency room with hyperglycemia six weeks after recovery from coronavirus disease 2019 (COVID-19) pneumonia. Laboratory results showed severe hyperglycemia, metabolic acidosis, positive ketones in urine and blood, and elevated fasting C- peptide level. COVID-19 polymerase chain reaction (PCR) was negative, and immunoglobulin G (IgG) antibodies were positive. The workup was completely unremarkable for acute infection. Hemoglobin A1C increased from 6.1% to 10.8% within six weeks. The mechanism by which COVID-19 infection may trigger the onset of full-blown diabetes mellitus remains unknown. Viral infection may cause the direct destruction of pancreatic beta cells or trigger the changes in the body that induce the state of insulin resistance. Antibodies against severe acute respiratory syndrome coronavirus 2 (SARS-CoV-2) infection may cross-react or interfere with the functioning of endogenous insulin. The association between type II diabetes and COVID-19 infections needs additional investigations to ascertain the exact mechanism by which COVID-19 infection triggers the onset of full-blown diabetes mellitus.

## Introduction

Diabetes mellitus is categorized into type I and type II. Type I is an insulin-dependent diabetes mellitus and is associated with severe deficiency of insulin due to the destruction of pancreatic beta cells, whereas type II diabetes is typically associated with increased insulin resistance, beta-cell exhaustion, and relative insulin deficiency [1]. Diabetic ketoacidosis is one of the most severe complications of diabetes mellitus and is typically associated with type I diabetes mellitus, but it can be seen with type II diabetes mellitus under the conditions of extreme stress including infections, trauma, emergencies, and sometimes as a presenting manifestation of ketosis-prone type II diabetes mellitus [2]. Several cases of patients infected with coronavirus disease 2019 (COVID-19) presented with severe hyperglycemia along with ketoacidosis or hyperosmolar-hyperglycemic state during their acute phase of illness have been identified [3]. In our case, the patient remained mildly hyperglycemic during the course of his COVID-19 pneumonia; however, he presented with diabetic ketoacidosis six weeks after being discharged.

## Case presentation

**Figure 1:**
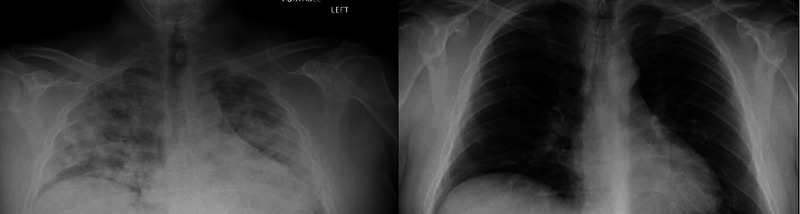
Chest X-ray on the left shows bilateral pneumonia, and chest X-ray on right shows clearance of pneumonia.

**Figure 2:**
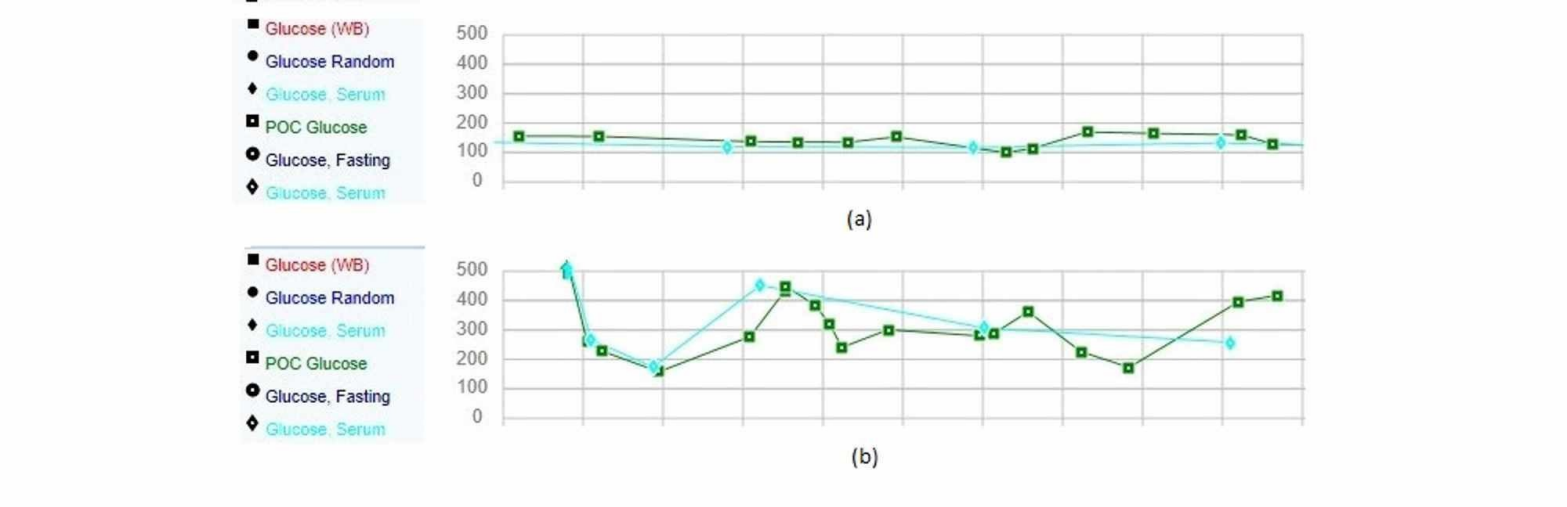
Blood sugar level during the first admission. (b) Blood sugar level during the second admission.

A 38-year-old male with a past medical history of prediabetes was hospitalized and treated for acute hypoxemic respiratory failure secondary to COVID-19 pneumonia. During that admission, blood sugar level remained below 150 mg/dL even while the patient was being administered glucocorticoids. Glucocorticoids were discontinued after five days of hospitalization. Six weeks later, he was admitted for a severe hyperglycemic state of glucose level over 500 mg/dL. Symptoms reported included polyuria and polydipsia for one week. Physical examination and vital signs were unremarkable except for a body mass index of 29.52 kg/m^2^. Initial labs revealed sodium of 124 mmol/L (normal range: 136-145 mmol/L), chloride of 86 mmol/L (normal range: 98-108 mmol/L), blood sugar of 532 mg/dL (normal range: 74-110 mg/dL), bicarbonate of 14 mmol/L (normal range: 22-29 mmol/L), pH of 7.27 (normal range: 7.32-7.42), anion gap of 24 mEq/L ( normal range: 8-16 mEq/L), and lactate 3.1 of mmol/L (normal range: 0.5-2.2 mmol/L). Serum and urine ketones were positive. COVID-19 polymerase chain reaction (PCR) test was negative, whereas COVID-19 immunoglobulin G (IgG) antibodies titer was positive. Urine and blood cultures were negative, and chest X-ray was negative for any pneumonia, as shown in Figure 1. Hemoglobin A1c was found to be 10.8% (normal range: 4-5.6%), whereas during the last admission with COVID-19 pneumonia hemoglobin A1c was found to be 6.1%. Labs further showed triglyceride level of 1,269 mg/dL (normal range: 10-149 mg/dL), fasting C-peptide level of 1.8 ng/mL (normal range: 1.1-4.4 ng/mL), and weakly positive glutamic acid decarboxylase antibodies. Islet cell antibodies, insulin antibodies, and zinc transporter 8 antibodies were negative. The patient was treated for diabetic ketoacidosis with insulin detemir as a basal insulin and insulin lispro as a bolus. The anion gap closed and blood sugar level showed improvement. The patient was later discharged on metformin and pioglitazone in addition to the basal-bolus insulin. The patient was followed for three weeks after the second discharge and reported fasting blood sugars in the range of 120 to 130 mg/dL with dietary modification, lifestyle changes, oral antidiabetics, and insulin (Figure 2).

## Discussion

The association between type II diabetes mellitus and viral infections has not been strongly established in the literature. Different viral infections including mumps, measles, echovirus, Epstein-Barr virus (EBV), and coxsackievirus have been traditionally associated with the onset of type I diabetes mellitus [4-8]. In one study, three patients, who developed ketosis-prone diabetes mellitus immediately after documented viral infection with EBV or coxsackievirus and required insulin therapy on discharge, were followed for four to eight years; the first patient remained insulin-dependent throughout the life, the second one regained normal glucose tolerance, and the third one was being transitioned to oral antidiabetic medications [6]. The study suggested that viral infections can trigger the onset of both type I and type II diabetes mellitus. Patients with COVID-19 infection who presented with diabetic ketoacidosis or hyperosmolar hyperglycemic state during the acute phase of infection have been identified [3,9]. One retrospective study demonstrated that out of 658 patients hospitalized with confirmed COVID-19 infection, 42 (6.3%) patients presented with ketosis, whereas 5 (0.76%) patients presented with ketoacidosis. Out of these five patients, three had established the diagnosis of diabetes and two were not previously diabetic [3]. This shows that COVID-19 infection causes physiological changes that induce ketosis irrespective of the presence or absence of diabetes. During the acute phase of infection, it is challenging to establish a cause and effect relationship between COVID-19 infection and diabetes mellitus.

This case is unique in that our patient with a history of prediabetes remained mildly hyperglycemic during acute phase of COVID-19 infection, only to develop diabetes mellitus after the acute phase of viral infection. The rise in hemoglobin A1c from 6.1% to 10.8% in just six weeks suggests that the blood sugar level trended up shortly after the first discharge from the hospital. The timeline for the onset of type II diabetes mellitus coincides with the recovery from COVID-19 infection. The mechanism by which COVID-19 infection may have triggered the onset of full-blown type II diabetes mellitus is yet to be understood. The viral infection may have caused the direct destruction of pancreatic beta cells [10] or triggered the changes in the body that induced the state of insulin resistance. Another hypothesis is that antibodies against severe acute respiratory syndrome coronavirus 2 (SARS-CoV-19) infection may cross-react or interfere with the functioning of endogenous insulin. This warrants further investigations to explain the association between COVID-19 infection and the onset of full-blown diabetes.

## Conclusions

We reported a case of a young male who developed full-blown ketosis-prone type II diabetes mellitus status post-SARS-CoV-2 infection recovery. Further studies are warranted to better understand the etiology and the pathophysiology of type II diabetes mellitus secondary to COVID-19 infection.

## References

[1] American Diabetes Association (2020). Diagnosis and Classification of Diabetes Mellitus. Diabetes Care.

[2] Smiley D, Chandra P, Umpierrez GE (2011). Update on diagnosis, pathogenesis and management of ketosis-prone Type 2 diabetes mellitus. Diabetes Manag (Lond).

[3] Li J, Wang X, Chen J, Zuo X, Zhang H, Deng A (2020). COVID‐19 infection may cause ketosis and ketoacidosis [Online ahead of print]. Diabetes Obes Metab.

[4] Makino K, Nishimae I, Suzuki N, Nitta S, Saitoh H, Kasao M, Takazawa K (2013). Myocarditis with fulminant type 1 diabetes mellitus diagnosed by cardiovascular magnetic resonance imaging: a case report. BMC Res Notes.

[5] Goto A, Takahashi Y, Kishimoto M, Nakajima Y, Nakanishi K, Kajio H, Noda M (2008). A case of fulminant type 1 diabetes associated with significant elevation of mumps titers. Endocr J.

[6] Nonaka K, Toyoshima H, Namba M, Tarui S (1982). Various phenotypes of diabetes mellitus at ultimate outcome of acutely developed diabetic state induced by viral infection. Endocrinol Jpn.

[7] Onal ED, Polat B, Balkan F, Kaya G, Ersoy R, Cakır B, Deniz O (2012). Positive measles serology and new onset of type 1 diabetes presented with bilateral facial paralysis: a case report. Braz J Infect Dis.

[8] Hiramatsu S, Komori K, Mori E, Ogo A, Maruyama S, Kato S (2011). A case of fulminant type 1 diabetes mellitus accompanied by myocarditis. Endocr J.

[9] Reddy PK, Kuchay MS, Mehta Y, Mishra SK (2020). Diabetic ketoacidosis precipitated by COVID-19: a report of two cases and review of literature. Diabetes Metab Syndr.

[10] Singh AK, Gupta R, Ghosh A, Misra A (2020). Diabetes in COVID-19: Prevalence, pathophysiology, prognosis and practical considerations. Diabetes Metab Syndr.

